# Comment on Barbieri et al. Umbilical Vein Blood Flow in Uncomplicated Pregnancies: Systematic Review of Available Reference Charts and Comparison with a New Cohort. *J. Clin. Med.* 2023, *12*, 3132

**DOI:** 10.3390/jcm13082347

**Published:** 2024-04-18

**Authors:** Jonathan M. Rubin, Oliver D. Kripfgans

**Affiliations:** Department of Radiology, University of Michigan, Ann Arbor, MI 48109-2026, USA; greentom@umich.edu

We would like to comment on the systemic review article published in the Journal of Clinical Medicine by Barbieri et al. Umbilical vein blood flow in uncomplicated pregnancies: systematic review of available reference charts and comparison with a new cohort [1].

We have been following the authors’ efforts in promoting the use of blood volume flow for assessing fetal well-being for over 20 years [2,3]. We agree that these authors have presented many reasons justifying the use of true umbilical cord volume flow estimates for fetal assessment [1,4]. In fact, it is very odd, that surrogate flow measures such as blood flow velocities or indices, such as systolic/diastolic ratios, are employed at all when true flow estimates are and have been available on most ultrasound machines for over four decades. However, as the authors point out, volume flow estimates are generally only employed in research settings [1]. This is despite the fact the ultrasound equipment and images have steadily improved with time. One must conclude that it is likely that there are systematic problems with the methodology that are at fault. Either the techniques Barbieri et al. [1] use fail because of incorrect assumptions and/or the measurements themselves are too cumbersome to perform or to inaccurate to be clinically useful. We believe that both are the case.

The method presented by Barbieri et al. [1] where volume flow in the umbilical vein (UV-Q) = mean velocity times (½ diameter)^2^, is calculated from b-mode images of the vessel and 1D sampling of the velocity using spectral Doppler. The estimates rely on several critical assumptions that in general are rarely if ever valid [5,6]. First, they assume that the flow in the umbilical vein is laminar and cylindrically symmetric. Given the spiral geometry of the vessels in the umbilical cord, this is almost never the case. In fact, the flow must be asymmetric with the higher velocities along the greater curvature of the vein [7]. Therefore, sampling for mean velocity estimates will vary depending on how the sample volume is positioned through the center of the vessel which is also consistent with the authors’ statement that volume flow estimates vary depending on the sampling site in the cord. Physically, this cannot be the case; since blood flow in the cord must be the same throughout its entire length since there are no sources or sinks that alter the flow [8]. More precise measurements in the free-floating cord than in the fetal abdomen must reflect systematic differences in the flow profiles or in the definitions of the vessel walls between the two sites. Further, assuming a Reynolds number for blood flow in the umbilical vein of about 350, even if the cord “looks straight”, a cylindrically symmetric profile will not have developed within the apparent straight segment due to prior turbulence or tortuosity [9]. Next, these estimates require angle correction, which is another source of error and an annoyance. Interestingly, some sampling methods suggest that scanning directly along the path of the vessel, which corresponds to a Doppler angle of zero degrees, can overcome this problem [10]. Typically, investigators then use ½ the peak velocity to represent the mean. This estimate again relies on the flow being laminar and cylindrically symmetric, which as pointed out, is rarely ever true [11]. Finally, vessel cross-sectional estimates are typically made using linear estimates of the vein diameter on b-mode images. This measurement assumes that the venous cross-section is a circle, a major assumption. Also, the measurement error is compounded by the fact that diameter is being used as a means of estimating cross-sectional area. Even if the cross-section is a circle, the relative error in the area estimate is twice the relative error in the diameter. So, for example, if an umbilical vein measures 4 mm in diameter, and the error is ±1 mm, the area estimate will have an error of ±50%. Lastly, the authors propose measuring the venous diameter from the back of the echo from the near wall to the front of the echo from the far wall. This measurement will be highly dependent on the transducer frequency and axial point-spread function. A better, less equipment dependent measure would be from the front of the front wall reflection to the front of the backwall reflection. All the sources of error mentioned above are systematic. Different methods of random error analysis such as z-scores will not solve these problems.

Because of these issues, we have been using flux estimates across blood vessels using Gauss’s divergence theorem to estimate volume flow [12,13,14,15]. This method has many advantages including angle independence, flow profile independence, and vessel geometry independence. Annoying measurements such as angle correction and diameter estimates are not required. The measurement process could be easy to perform and nearly automatic. We have shown about a 2.5 times improvement in precision of umbilical vein flow measurements using this method over the spectral Doppler technique the authors use [15]. The method uses standard color and power flow ultrasound with one additional requirement; it must be done in 3D. A standard 3D mechanical probe used for making baby faces will work. Using a 2D array probe, literally dozens of estimates can be obtained in a few seconds, making mean flow measurements statistically very robust.

The biggest obstacle to general clinical implementation of this method is that unlike standard spectral Doppler, it has not been implemented on any clinical ultrasound machines. This is unfortunate since any ultrasound machine that can carry out 3D imaging along with color flow and color power imaging can implement this new method. We would hope that these authors, along with other practitioners interested in estimating umbilical venous volume flow, would encourage ultrasound companies to incorporate this method on their machines. Hopefully, we will see a new leap in the levels of fetal assessment initiated by measuring true blood flow in the umbilical cord.
